# Patients with severe schistosomiasis mansoni in Ituri Province, Democratic Republic of the Congo

**DOI:** 10.1186/s40249-021-00815-6

**Published:** 2021-03-25

**Authors:** Maurice M. Nigo, Peter Odermatt, David Wully Nigo, Georgette B. Salieb-Beugelaar, Manuel Battegay, Patrick R. Hunziker

**Affiliations:** 1 Nanomedicine Translation Group, Intensive Care Unit, University Hospital Basel University of Basel, Petersgraben 4, 4031 Basel, Switzerland; 2 CLINAM-European Foundation for Clinical Nanomedicine, Alemannengasse 12, P.O. Box, 4016 Basel, Switzerland; 3 University of Basel, Petersplatz 1, Basel, Switzerland; 4 Institut Supérieur Des Techniques Médicales (ISTM) Nyankunde, BP 55 Bunia, Democratic Republic of Congo; 5 Swiss Tropical and Public Health Institute, Socinstrasse 57, P.O. Box, 4002 Basel, Switzerland; 6 Centre Hospitalier, Ingbokolo, Democratic Republic of Congo; 7 Department of Infectiology and Hospital Hygiene, University Hospital Basel, Petersgraben 4, 4031 Basel, Switzerland

**Keywords:** Intestinal schistosomiasis, Severe case, Hepatomegaly, Splenomegaly, Ascites, Hematemesis, Morbidity, Mortality, Democratic Republic of the Congo, Ultrasonography

## Abstract

**Background:**

Severe hepatosplenic complications arise in patients with chronic *Schistosoma mansoni* infection after heavy exposure to disease agents in endemic areas. These complications are rarely reported and, hence, underestimated.

**Case presentation:**

We report on eight patients with severe morbidity associated with *S. mansoni* infection in Ituri Province, northeastern Democratic Republic of Congo (DRC). The patients were identified during a community-based survey in 2017; one patient was seen at the district hospital. After taking the patients’ history, a clinical examination and an abdominal ultrasonographical examination were performed. *S. mansoni* infection was diagnosed in fecal (Kato-Katz technique) and urine (point-of-case circulating cathodic antigen test) samples. These eight patients with severe intestinal and hepatosplenic complications were identified from four villages with high *S. mansoni* infection prevalence and related morbidity. The patients’ ages ranged from 19 to 57 years; four patients were women. Three patients reported hematemesis. Two patients were severely anemic. All patients reported non-specific abdominal symptoms, such as diarrhea (six patients), abdominal pain (seven patients), and blood in the stool (five patients), as well as weight loss (two patients). Abdominal ultrasonography revealed ascites in four patients. All patients had portal hypertension with hepatomegaly (seven patients) or splenomegaly (five patients). Of the six patients with a discernable liver parenchyma pattern, five displayed pattern F and three patient displayed pattern E. Liver parenchyma was not visible for two patients with severe ascites. An *S. mansoni* infection was confirmed in six patients, with infection intensity ranging from light to heavy. All *S. mansoni* positive patients were treated with praziquantel (40 mg/kg body weight) and referred to the district hospital for follow-up. One patient with severe ascites died two weeks after we saw her. Due to security and accessibility reasons, the villages could not be visited again and the patients were lost to follow-up.

**Conclusions:**

Our observations of patients with severe schistosomiasis document the severe degree of endemicity of *S. mansoni* in the province and suggest an urgent need for adequate schistosomiasis control measures that target vulnerable population groups and address severe complications.
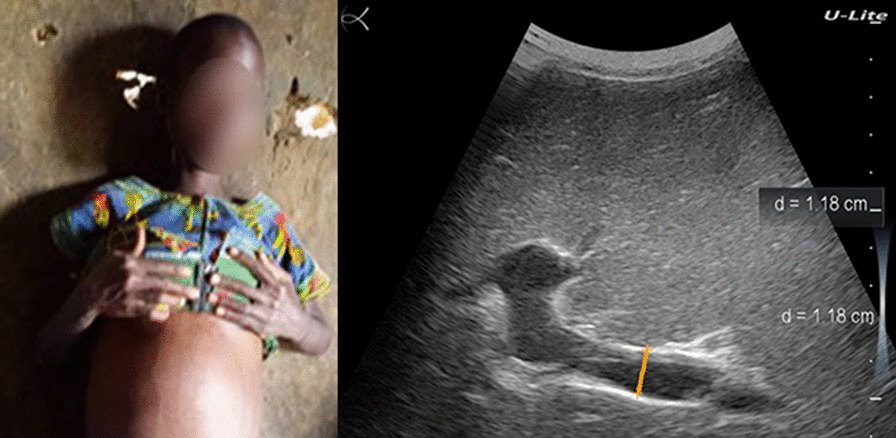

**Supplementary Information:**

The online version contains supplementary material available at 10.1186/s40249-021-00815-6.

## Background

Schistosomiasis is a widespread, neglected, and debilitating disease [1]. *Schistosoma* infections afflict an estimated 220 million people worldwide, 90% of whom live in sub-Saharan Africa [2]. The morbidity and mortality associated with *Schistosoma* infection represents a major public health problem.

In sub-Saharan Africa, *S. mansoni* is a very common factor in up to 60% of patients with hematemesis [3]. Symptoms of chronic infection may include abdominal pain, diarrhea, bloody stools, anemia, weight loss, and stunting. Hepatomegaly and splenomegaly indicate a severe hepatosplenic pathology, including portal hypertension.

For more than three decades, the World Health Organization (WHO) has promoted preventive chemotherapy using the anti-helminthic drug, praziquantel, to control morbidity and mortality due to *Schistosoma* infection. Implementing preventive chemotherapy poses substantial operational challenges, particularly in low-income countries with inadequate public health resources. Regular (annual) treatment of school-aged children is the most common activity. However, in many schistosomiasis-endemic settings, it is an insufficient measure for controlling morbidity and mortality.

Although *Schistosoma mansoni* infection is widespread in the Democratic Republic of Congo (DRC), relatively little recent information is available about the disease burden associated with the infection, particularly in the rural parts of the country. Malaria is highly endemic in the country. Viral, bacterial, protozoon, and helminth co-infections are also very frequent [4].

Between 2015 and 2017, three large cross-sectional studies were conducted in Ituri Province, in northeastern DRC. Both the prevalence of *S. mansoni* infection and its associated morbidity were found to being high in the province [5, 6].

Here, we describe eight patients with severe complications due to *S. mansoni* infection in Ituri Province, Democratic Republic of Congo, and document the level of endemicity of schistosomiasis in the country.

## Case presentation

Patients were identified during a community-based survey conducted between June and September, 2017. One patient presented at the Angumu hospital. The patients were clinically assessed and interviewed. Their general health concerns and history were assessed (Table 2). An abdominal ultrasound was performed, and a stool sample was examined for helminth infections.

### Characteristics of patient village environments

Ituri Province is situated in northeastern DRC and has a surface area of 65 658 km^2^. With its capital in Bunia, Ituri is divided into five counties (territories) and 36 health districts. About 5.3 to 9.0 [7] million people of Sudanese, Nilotic, Bantu, Nilo-Hamite, and Pygmy ethnicities live in Ituri province.

The patients we report on here originate from four villages: Gupe and Ndaro, in the Angumu health district located on the shore of Lake Albert, east of Bunia; and Mandima and Pekele, in the Mandima and Lolwa health districts, respectively. The latter two villages are situated opposite each other, on the shores of a Maholanzoka stream in southern Ituri, approximately 160 km west of Bunia and approximately 200 km from Lake Albert. *S. mansoni* transmission around Lake Albert has been documented since colonial times [11]. A recent study showed intensive transmission of *S. mansoni* in the villages around the lake [5], as well as in Mandima and Pekele.

The four villages were part of a large in-depth study of morbidity due to *S. mansoni* infection. The complete results of the study are available elsewhere [6]. Table 1 is an excerpt from the study, describing the epidemiological context of the four villages in which the patients with severe cases resided. The villages are characterized by very high *S. mansoni* prevalence [74.3%, as assessed with a Kato-Katz test alone, and 86.6% when Kato-Katz results were combined with point-of-care circulating cathodic *S. mansoni* antigen (POC-CCA) test results] and a high-intensity infection burden (15.9% had a heavy-intensity infection). Soil-transmitted helminths were almost absent, indicating that regular deworming with benzimidazoles had been conducted. More than half (56.1%) of the population was underweight. Intestinal morbidity (e.g., 59.8% with abdominal pain) and hepatosplenic morbidity (e.g., 42.1% with splenomegaly, 31.1 with hepatomegaly, and 71.3% with abnormal liver parenchyma) was frequent.

**Table 1:** *Schistosoma mansoni* infection and associated morbidity in the four villages where the eight patients resided in 2017 (*n* = 164)

Characteristics	*n*	%
Gender		
Females	87	53.0
Males	77	47.0
Age categories (years)		
6–9	32	19.5
10–14	40	24.4
15–19	19	11.6
20–29	17	10.4
30–39	22	13.4
≥ 40	34	20.7
Body mass index (kg/m^2^—categories)		
Overweight (≥ 25.0)	10	6.1
Normal weight (18.5–24.9)	62	37.8
Underweight (< 18.5)	92	56.1
*S. mansoni* infection		
Kato-Katz test	118	72.0
POC-CCA test	129	78.7
KK + POC-CCA*	142	86.6
Infection intensity (KK only)		
Negative	46	28.0
Light	51	31.1
Moderate	41	25.0
Heavy	26	15.9
Clinical findings		
Diarrhea	56	34.2
Blood in stool	56	34.2
Abdominal pain	98	59.8
Ultrasound findings		
Hepatomegaly	51	31.1
Splenomegaly	69	42.1
Ascites	4	2.4
Pattern A	47	28.7
Pattern B	14	8.5
Pattern C	61	37.2
Pattern D	18	11.0
Pattern E	13	7.9
Pattern F	11	6.7

*KK + POC-CCA, combined any positive result (by Kato-Katz and/or by point-of-care circulating cathodic antigen [POC-CCA]); KK only, only Kato-Katz test results taken into account (at least one egg in at least one of two smears). Liver patterns associated with schistosomiasis: A normal; B starry sky; C rings and pipe-stems; D ruff around portal bifurcation; E patches; F bird’s claw

### Patient assessment

#### Clinical examination

All participants underwent a clinical (physical) examination and a questionnaire-guided interview, both of which were conducted by an experienced physician with assistance from an experienced nurse.

Pre-tested individual and household questionnaires (see Additional file 1, Additional file 2), written in French and translated into Kiswahili or other appropriate local language, were administered in the patient’s household. The household questionnaire focused on the immediate household environment: sanitation, water sources, proximity to a body of water, and distance from the health center. The individual questionnaire focused on demographic, anthropometric, occupational, educational, and religious characteristics, as well as on knowledge, attitude, and practices related to *S. mansoni* infection and disease. It also included an assessment of signs and symptoms related to schistosomiasis, such as diarrhea, blood in the stool in the two weeks preceding the study, and individual history of hematemesis at least once in their lifetime.

#### Abdominal ultrasound examination

An abdominal ultrasonographical examination was performed in accordance with the WHO/TDR guidelines [12], using a 1.0 MHz probe U-Lite Sonoscanner Ultraportable HD Ultrasound Unit (U-Lite, Sonoscanner, 6, Rue André Voguet, Paris, France). A portable generator (MK, China) and solar powered batteries (for remote villages) were used as electricity sources.

Participants were examined in a supine position. The size of the left and right liver lobe, the portal vein diameter, and the length and width of the gall bladder were measured. Organ parenchyma was observed. Liver parenchyma patterns (Additional file 3: Figure S1) were assessed following the WHO/TDR guidelines [12]. The length and width of the spleen were measured, and its texture determined.

#### Parasitological examination

Participants were asked to provide one fecal sample (approximately 5 g of morning stool) for a Kato-Katz test [13]. Labelled plastic containers were provided for collection. From each stool specimen, two thick smears of approximately 41.7 mg [13] were prepared and examined by experienced technicians. For hookworm assessment, the smears were examined by microscope within one hour of preparation. All slides were examined for *S. mansoni* within 24 h. One third of the prepared smears were checked by the principal investigator. All recognized helminth eggs were counted and recorded for each species separately. The intensity of helminth infection was calculated by multiplying the mean number of eggs found on the two slides by 24. The result was expressed as EPG of stool [14]. *S. mansoni* infection intensities were classified as light (1–99 EPG), moderate (100–399 EPG), or heavy (≥ 400 EPG) [14].

Participants were asked to provide a urine sample (in a vial of approximately 60 ml) for a point-of-care circulating cathodic antigen (POC-CCA) test. Pre-labelled wide-mouth plastic containers were provided for urine collection. The POC-CCA tests were performed according to the manufacturer’s guidelines (Rapid Medical Diagnostics, Pretoria, South Africa) [15]. Urine was examined on the day of collection where possible. If the test was postponed until the next day, urine samples were kept at 2–8 ℃ in a solar fridge (Steca, Germany). A negative test result was declared if the POC-CCA band did not appear within 20 min. A positive result was recorded for any trace-, weak-, medium- and strong-colored CCA bands. Questionable results were discussed among at least two technicians and the principal investigator. Both stool and urine examinations were performed at village health center facilities.

#### Hemoglobin dosage

For two participants with extreme palpebral conjunctiva pallor, hemoglobin rates were estimated at the Angumu hospital laboratory, using a Sahli hemoglobinometer. Results were expressed as gram of total hemoglobin per liter of blood (g/L). Normal hemoglobin ranges are situated between 120.0 and 155.0 g/L in women and 135.0–175.0 g/L in men. Thus, a hemoglobin rate < 120.0 g/L was considered anemic. Using the two cut-off points of hemoglobin measurement as recommended by WHO for Africans [16], anemia is considered ‘light’ if the hemoglobin rate is between 90.0 and 119.0 g/L, and severe if the hemoglobin rate is < 90.0 g/L.

Participants diagnosed with *S. mansoni* were treated with praziquantel (40 mg/kg) [17]. All participants received mebendazole (500 mg, single dose) for general deworming, in accordance with the DRC’s national deworming guidelines.

### Patient descriptions

Characteristics of and medical information for the eight patients are given in Table 2. The patients’ ages ranged from 19 to 57 years (mean 35.9 years); four patients were females. Three patients (37.5%) experienced hematemesis, seven patients (87.5%) reported abdominal pain, six patients (75.0%) had chronic diarrhea, and five patients (62.5%) reported blood in the stool.

**Table 2:** Characteristics of and medical information for eight patients with severe schistosomiasis

Patient code	Age (years), gender	Occupation, education	*Schistosoma mansoni* infection	Symptoms/signs and medical history	Ultrasound	Treatment	Observations (outcome)
1	31, Male	Fisherman No formal education	Kato-Katz positive, severe infection intensity (2832 EPG), POC-CCA positive	Hematemesis, melena, anemia, tachycardia, exhausted No history of illness	Fibrotic liver pattern decreased definition of portal vein wall and a heterogeneous echotexture of liver parenchyma	Mebendazole 500 mg Praziquantel 40 mg/kg	Did not know schistosomiasis. Cured 5 days later
2	57, Female	Housewife, fish trader Primary school	Kato-Katz and POC-CCA negative	Anemia, abdominal pain, mucus in stool, weight loss, tachycardia, dyspnea, exhausted Reported previous severe illness	Ascites, distorted liver architecture, nodular surface, heterogeneous echotexture of liver parenchyma	Mebendazole 500 mg	Knew schistosomiasis Died two weeks later
3	51, Male	Fisherman No formal education	Kato-Katz negative; POC-CCA positive	Severe weight loss, abdominal pain, mucus in stool, tachycardia, exhausted, Reported previous severe illness	Ascites, distorted liver architecture, nodular surface, heterogeneous echotexture of liver parenchyma	Mebendazole 500 mg Praziquantel 40 mg/kg	Did not know schistosomiasis Lost to follow-up
4	36, Male	Fisherman Primary school	Kato-Katz positive, light infection intensity (36 EPG); POC-CCA positive	Hematemesis, abdominal pain, diarrhea, blood in stools Reported frequent episodes of ill health	Distorted liver architecture and heterogeneous echotexture of liver parenchyma, and decreased definition of portal vein wall	Mebendazole 500 mg Praziquantel 40 mg/kg	Knew schistosomiasis Lost to follow-up
5	22, Female	Housewife, fish trader No formal education	Kato-Katz positive, moderate infection intensity (240 EPG), POC-CCA positive	Hematemesis, abdominal pain, diarrhea, blood in stools, mucus in stool, exhausted Reported previous sporadic illness	Starry night sky liver pattern, liver architecture and heterogeneous echotexture of liver parenchyma, and decreased definition of portal vein wall	Mebendazole 500 mg Praziquantel 40 mg/kg	Did not know schistosomiasis Lost to follow-up
6	19, Male	Fisherman No formal education	Kato-Katz positive, light infection intensity (24 EPG); POC-CCA positive	Abdominal pain, diarrhea, blood in stools, mucus in stool, severe weight loss, stunting, exhausted Reported frequent episodes of unspecified sickness	Starry night sky liver pattern, liver architecture and heterogeneous echotexture of liver parenchyma, and decreased definition of portal vein wall	Mebendazole 500 mg Praziquantel 40 mg/kg	Did not know schistosomiasis Lost to follow-up
7	40, Female	Housewife, farmer No formal education	Kato-Katz positive, moderate infection intensity (156 EPG); POC-CCA positive	Abdominal pain, diarrhea, blood in stools, severe weight loss, mucus in stool, exhausted Reported severe (unknown) sickness in past	Ascites, liver with distorted architecture, nodular surface, and heterogeneous echotexture of the parenchyma	Mebendazole 500 mg Praziquantel 40 mg/kg	Did not know schistosomiasis Lost to follow-up
8	29, Female	Housewife, No formal education	Kato-Katz and POC-CCA negative	Abdominal pain, diarrhea, blood in stools, mucus in stool, severe weight loss, exhausted Reported severe (unknown) sickness in past	Ascites, liver with distorted architecture, nodular surface, heterogeneous and starry night sky echotexture of the parenchyma	Mebendazole 500 mg	Did not know schistosomiasis Lost to follow-up

Diagnosed in four purposively-selected villages in Ituri Province

*POC-CCA* point-of-care circulating cathodic *S. mansoni* antigen test

Abdominal ultrasonography revealed hepatomegaly in seven patients (87.5%), splenomegaly in six patients (62.5%), and ascites in four patients (50.0%). Three patients had pathologic liver parenchyma pattern E (37.5%), and five patients (62.5%) had pattern F.

*S. mansoni* infection was confirmed for six patients. Patients #2 and #8 tested negative with POC-CCA and negative with Kato-Katz. Infection intensities ranged from 24 to 2832 EPG (mean: 406 EPG). One heavy-intensity infection was diagnosed, while two patients each had moderate- and light-intensity infections. All patients were treated with praziquantel (40 mg/kg body weight). The hospitalized patient (patient #1) was discharged five days after treatment. One patient with severe ascites (patient #2) died two weeks after we saw her. Due to security concerns, the villages could not be visited for follow-up examinations. Hence, seven patients were lost to follow-up.

### Patient 1

A 31-year-old inveterate fisherman residing in Gupe village on the shore of Lake Albert (Angumu health district) had been actively fishing for several days. He was urgently referred to Angumu hospital following five days of ongoing blackish feces, and sudden vomiting of blood and dehydration on the day of admission. He showed altered general state and Hippocratic facies. However, he was lucid and had an athletic constitution. He continued to vomit blood. He had a history of alcohol addiction but had never been treated for schistosomiasis.

**Fig. 1:**
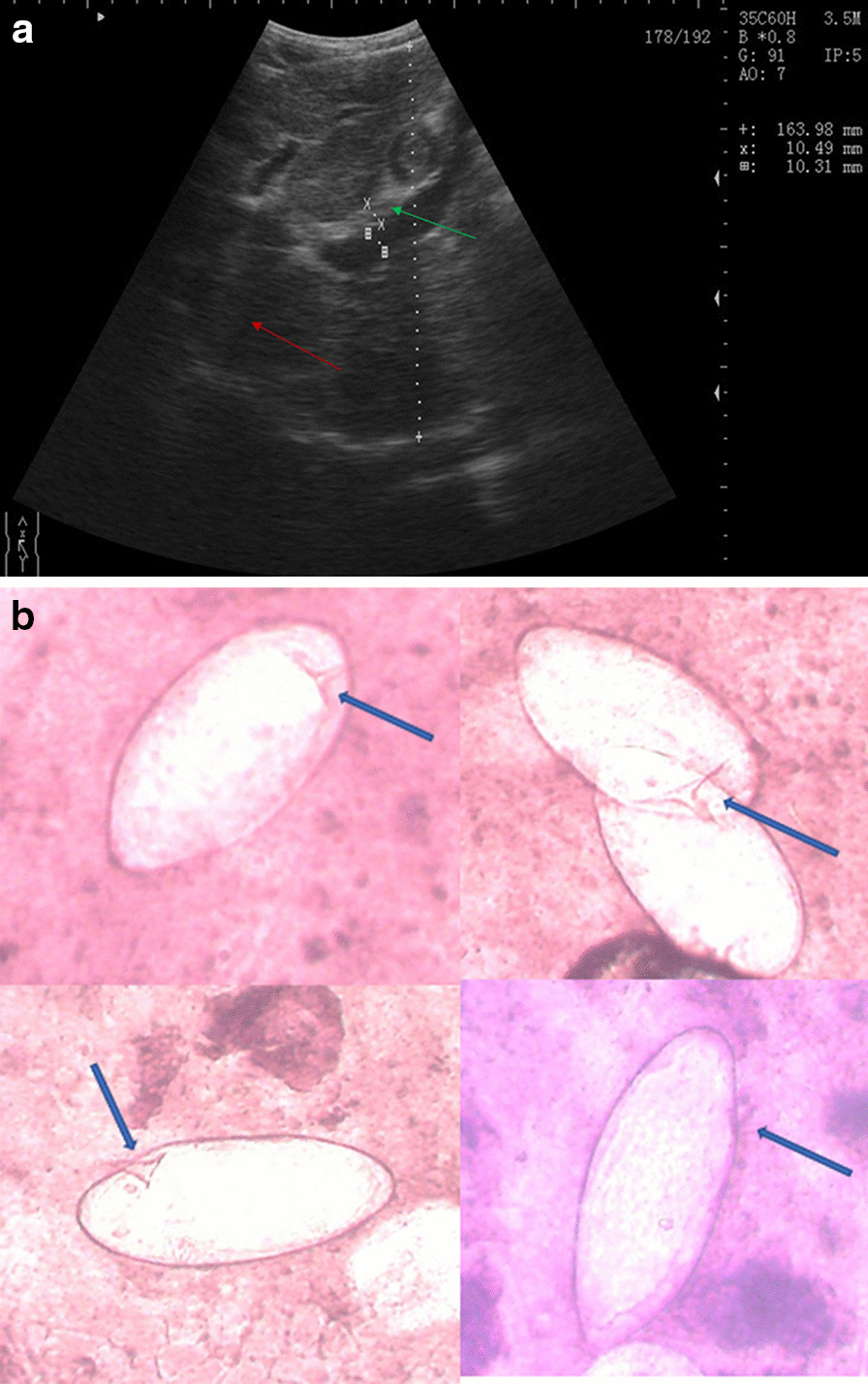
**a** Ultrasound examination of patient #1: fibrotic liver pattern showing decreased definition of portal vein wall (green arrow) and a heterogeneous echotexture of liver parenchyma (red arrow). **b** *Schistosoma mansoni* eggs in bloody stool (patient #1). Blue arrows show the eggs’ characteristic lateral spine

His physical examination was unremarkable. Although the abdomen showed epigastric tenderness, its percussion revealed no matting and, therefore, no ascites. The palpebral conjunctivae were pale and the bulbar conjunctivae were anicteric. Examination of the thorax revealed tachycardia, but normal respiration. Abdominal ultrasound revealed hepatomegaly and splenomegaly. The liver showed a fibrotic pattern, with decreased definition of the portal vein wall and a heterogeneous echotexture of the liver parenchyma (Fig. 1a).

He was severely anemic (hemoglobin 70.0 g/L). Direct microscopic examination of the brownish-blackish stool revealed the presence of *S. mansoni*'s side-spur eggs (Fig. 1b). A Kato-Katz stool examination showed severe infection intensity (2832 EPG). The POC-CCA was positive. The malaria rapid diagnostic test was negative.

Saline infusion (NaCl 9‰, 1 L) was administered, followed by 500 ml of a 5% glucose solution with vitamin K. The patient received a blood transfusion and was discharged five days after admission. The patient was treated with praziquantel.

### Patient 2

A 57-year-old woman from Gupe village (Lake Albert, Angumu health district), presented with a distended abdomen. Her general state was severely altered. She had a history of schistosomiasis and had been treated several times. Her physical examination was typical of patients with severe intestinal schistosomiasis. She had epigastric tenderness of the abdomen; abdominal percussion revealed matting and extended ascites. She was severely anemic (50.0 g/L). She also had tachycardia, respiratory distress, and tachypnoea due to the immense ascites.

**Fig. 2:**
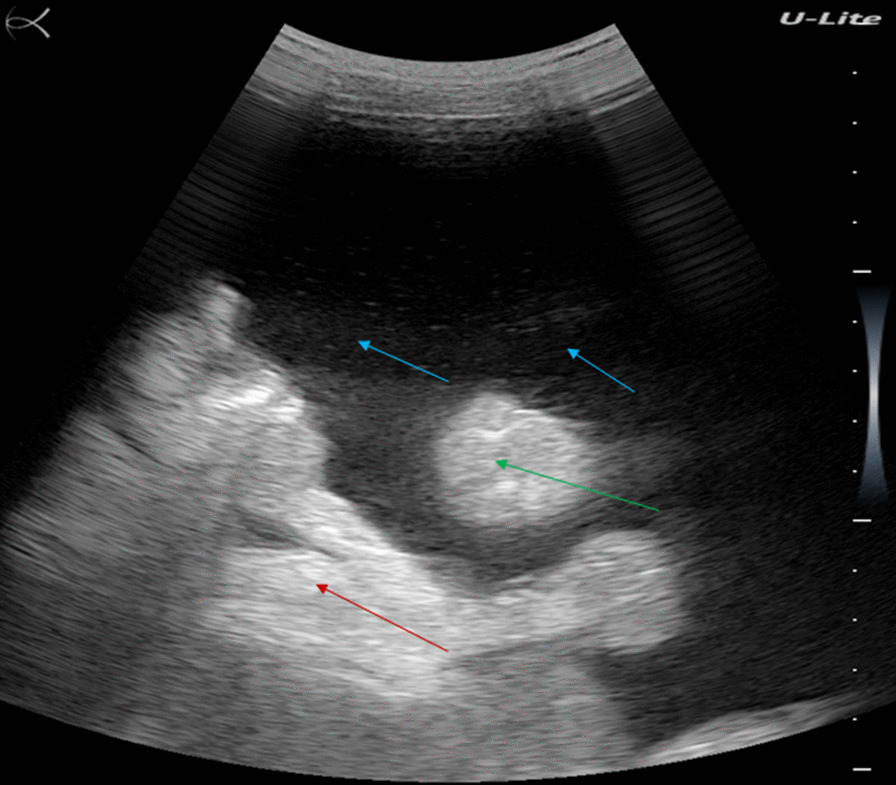
Ultrasound examination of patient #2, showing shrunken cirrhotic liver with distorted architecture, nodular surface, and heterogeneous echotexture of the parenchyma (red arrow), regenerating nodules (green arrow), and ascites (blue arrow)

Ultrasound examination revealed a shrunken cirrhotic liver with distorted architecture, nodular surface, and heterogeneous echotexture of the parenchyma, regenerating nodules, and ascites (Fig. 2). She also had splenomegaly.

Both stool and urine tests were negative for *S. mansoni* infection. The patient died two weeks later.

### Patient 3

**Fig. 3:**
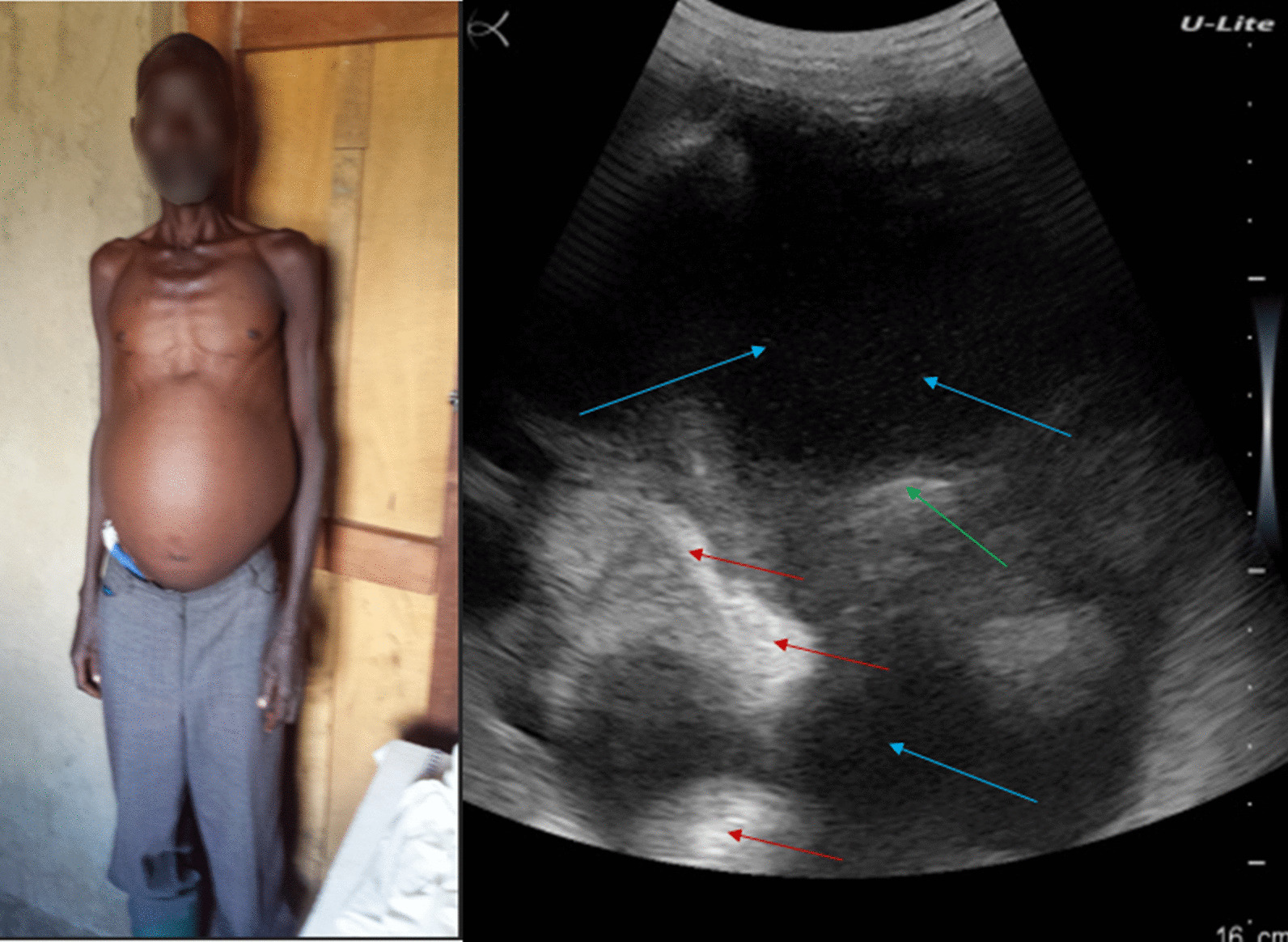
Patient #3 (left). Ultrasound examination (right) showing shrunken cirrhotic liver with distorted architecture, nodular surface, and heterogeneous echotexture of the parenchyma (red arrow), regenerating nodules (green arrow), and ascites (blue arrow)

A 51-year-old fisherman from Gupe village (Lake Albert, Angumu health district) presented with an distended belly. His general state was severely altered with anorexia, asthenia, and significant weight loss (Fig. 3, left). He was visibly tired but was alert and lucid. He had a history of schistosomiasis and had been treated several times some years before. He had worked as a fisherman on Lake Albert for many years.

His abdomen showed epigastric tenderness; abdominal percussion revealed matting, indicating extended ascites. Ultrasound examination revealed a shrunken cirrhotic liver with distorted architecture, nodular surface, and heterogeneous echotexture of the parenchyma, regenerating nodules, and ascites (Fig. 3, right). He also had splenomegaly.

A stool examination was negative for *S. mansoni* infection, but a POC-CCA was positive. The patient was treated with praziquantel. Three weeks after the visit, the patient’s sister took him to his native village. He was subsequently lost to follow-up.

### Patient 4

A 36-year-old man residing in the fishing village of Ndaro, on the shore of Lake Albert, in Angumu health district, complained of abdominal pain, diarrhea, and the presence of blood in the stool for some time. He had experienced hematemesis two times.

**Fig. 4:**
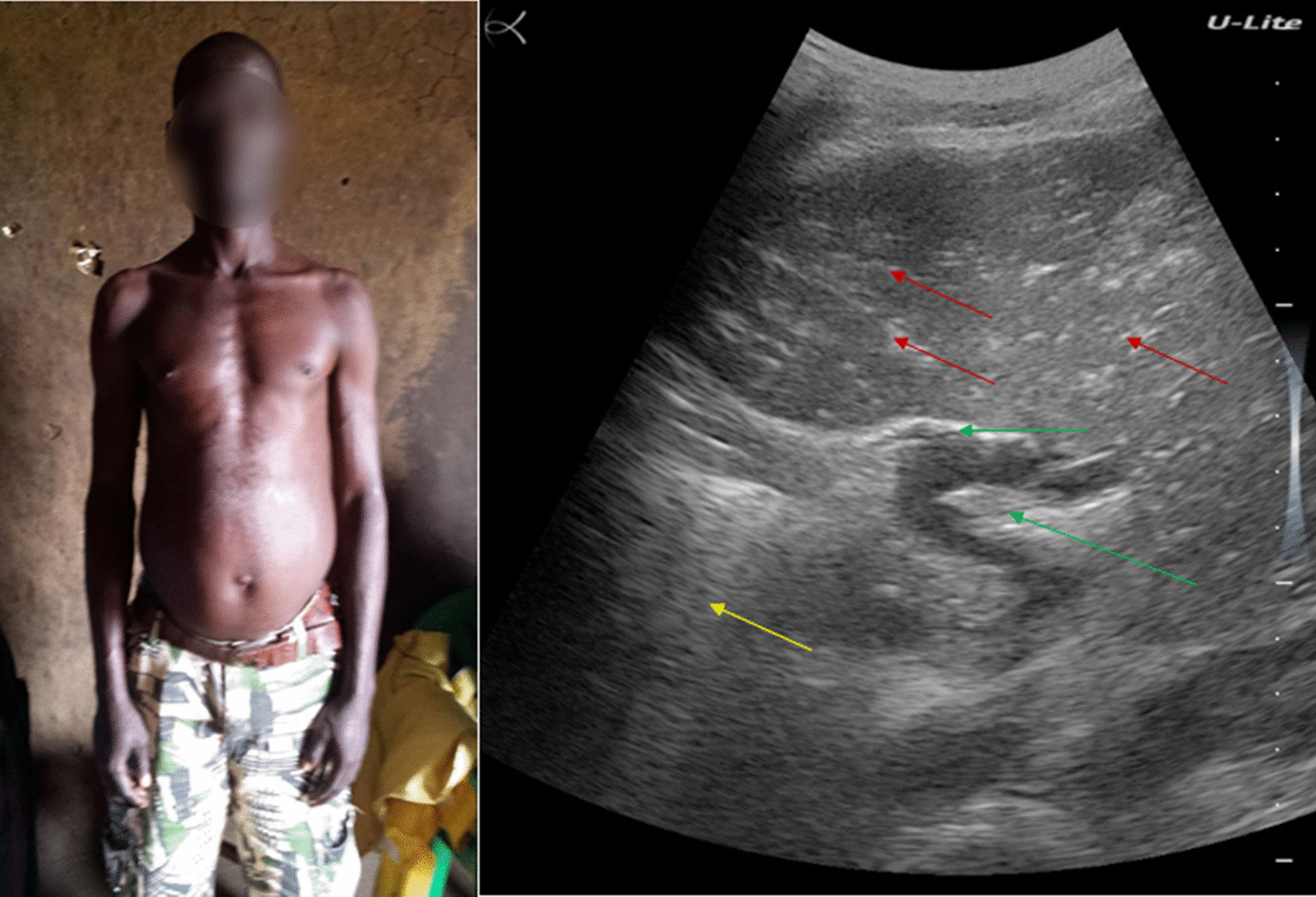
Patient #4 (left). Ultrasound examination (right) showing distorted liver architecture and heterogeneous echotexture of liver parenchyma and starry night sky liver pattern (red arrow), decreased definition of the portal vein wall (green arrow), and posterior reinforcement (yellow arrow)

His general state was quite good (Fig. 4, left). He had a history of schistosomiasis and had been treated twice. He had been working for several years as a fisherman.

His abdomen showed epigastric tenderness; however, abdominal percussion revealed no matting, thus, no ascites. Ultrasound examination showed a distorted liver architecture and heterogeneous echotexture of liver parenchyma and starry night sky liver pattern, decreased definition of the portal vein wall, and posterior reinforcement (Fig. 4, right). He also had splenomegaly.

Both diagnostic tests were positive for a light-intensity *S. mansoni* infection (36 EPG). He was treated with praziquantel and subsequently lost to follow-up.

### Patient 5

A 22-year-old woman who had lived since childhood in the fishing village of Ndaro (Lake Albert, Angumu health district), complained of abdominal pain, diarrhea, and the presence of blood in her stool for some time. She had vomited blood on one occasion. She firmly believed that she was dealing with wizards. She was married in her youth to a fisherman, for whom she became a fish trader. Her husband abandoned her when she became sick.

She appeared exhausted and could not recall having been treated before for schistosomiasis. She had been accompanying her husband on fishing trips before she became sick.

**Fig. 5:**
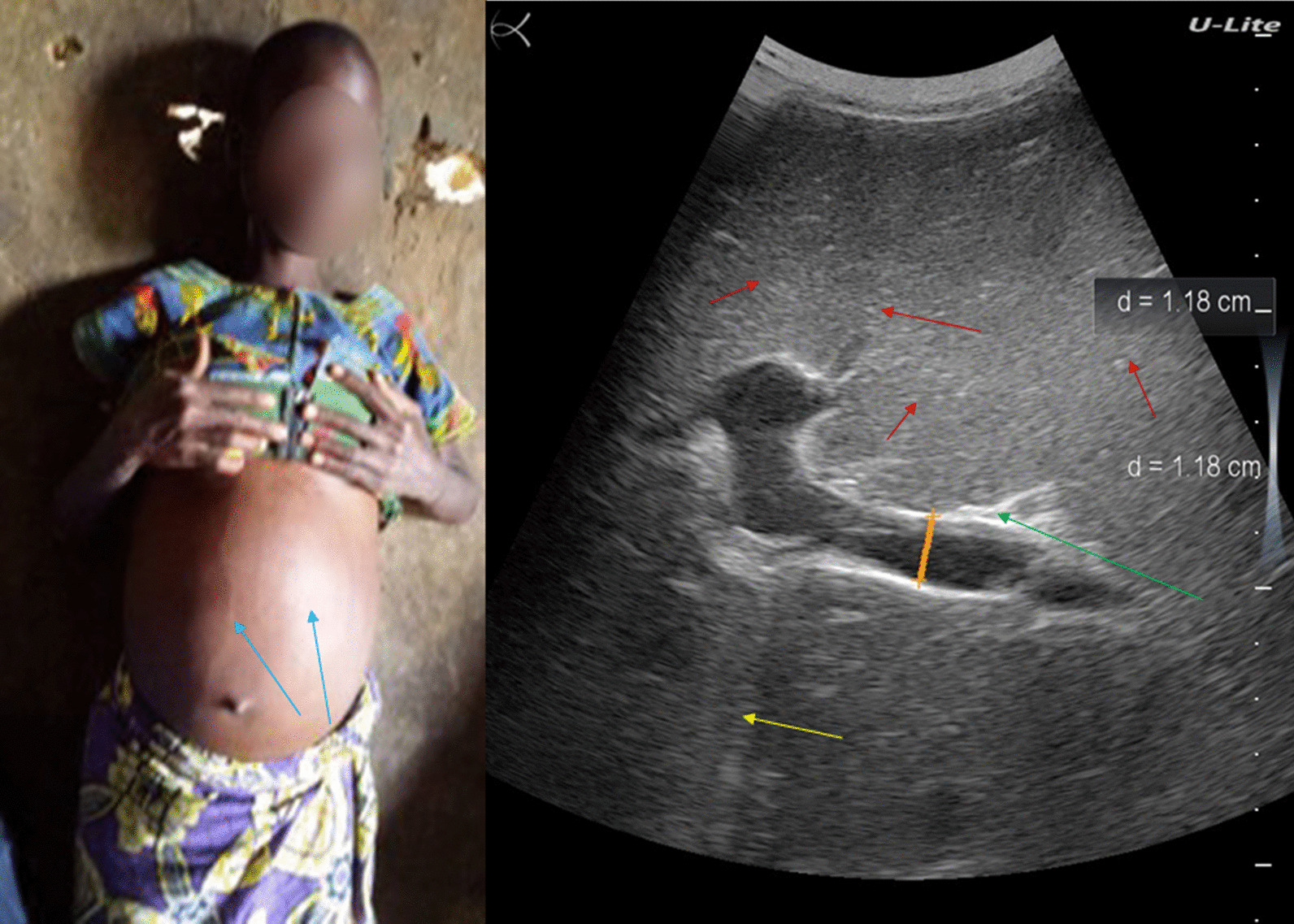
Patient #5 (left) showing collateral veins and large splenomegaly (blue arrows). Ultrasound examination (right) showing starry night sky liver pattern, liver architecture and heterogeneous echotexture of liver parenchyma (red arrow), decreased definition of portal vein wall (green arrow), and posterior reinforcement (yellow arrow)

Her abdomen was distended, showing collateral veins and large palpable splenomegaly (Fig. 5, left), and showed epigastric tenderness; however, percussion revealed no matting, thus, no ascites. Ultrasound examination showed a starry night sky liver pattern, liver architecture and heterogeneous echotexture of liver parenchyma, decreased definition of the portal vein wall, posterior reinforcement, and splenomegaly (Fig. 5, right).

Stool examination was positive for *S. mansoni* eggs, with a moderate infection intensity (240 EPG); POC-CCA was also positive. She was treated with praziquantel and subsequently lost to follow-up.

### Patient 6

**Fig. 6:**
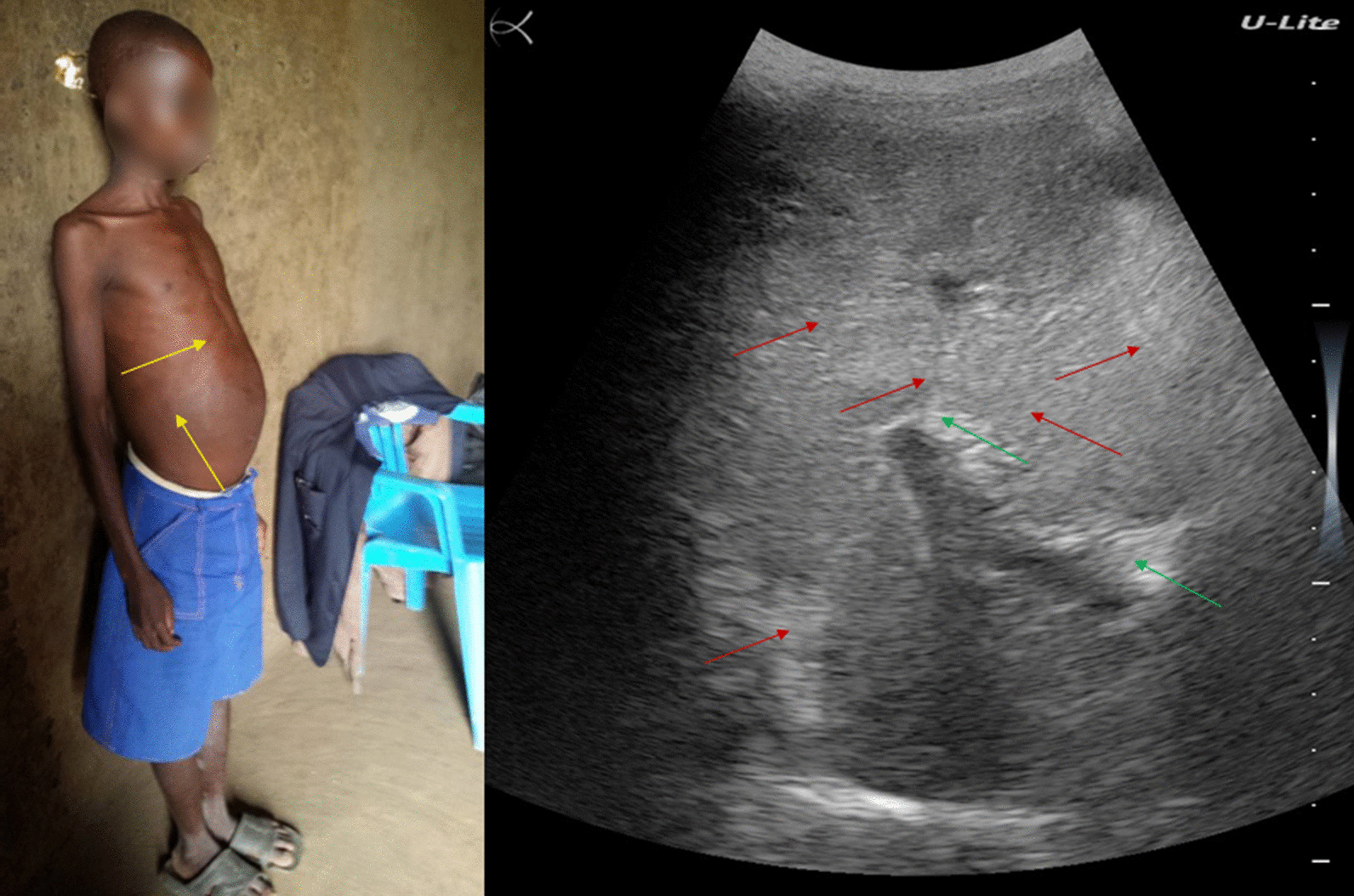
Patient #6, a cachectic young man with chronic rash spots (yellow arrows, left). Ultrasound examination (right) showing starry night sky liver pattern, liver architecture and heterogeneous echotexture of liver parenchyma (red arrow), and decreased definition of the portal vein wall (green arrow)

A 19-year-old fisherman had lived in the fishing village of Ndaro (Lake Albert, Angumu health district) since childhood. Apparently, he had been abandoned by his parents and lived in the village like a street child. He complained of abdominal pain, diarrhea, and the presence of blood in his stool. He had not vomited blood. He was tired and reported severe weight loss, which had reduced him to a weight of 38 kg at the time of examination. He was stunted (149 cm for a 19-year-old man), cachectic, and had a chronic rash (spots) on his abdomen (Fig. 6, left). He had a history of schistosomiasis and had been treated several times. He had been working as a fisherman already for many years.

His abdomen showed epigastric tenderness; however, percussion revealed no matting, thus, no ascites. Ultrasound examination showed a starry night sky liver pattern, liver architecture and heterogeneous echotexture of liver parenchyma (red arrow), decreased definition of the portal vein wall (Fig. 6, right), and splenomegaly.

He was diagnosed with a light-intensity *S. mansoni* infection. POC-CCA confirmed the infection. He was treated with praziquantel and subsequently lost to follow-up.

### Patient 7

A 40-year-old farming woman residing in the forest village of Mandima since birth (Mandima health district, southwest Ituri), complained of weight loss, fatigue, abdominal pain, and diarrhea, but no blood in stool. She had not vomited blood. She reported that she had not travelled to the Lake Albert region. The patient had never heard about schistosomiasis and had never been diagnosed nor treated for it.

**Fig. 7:**
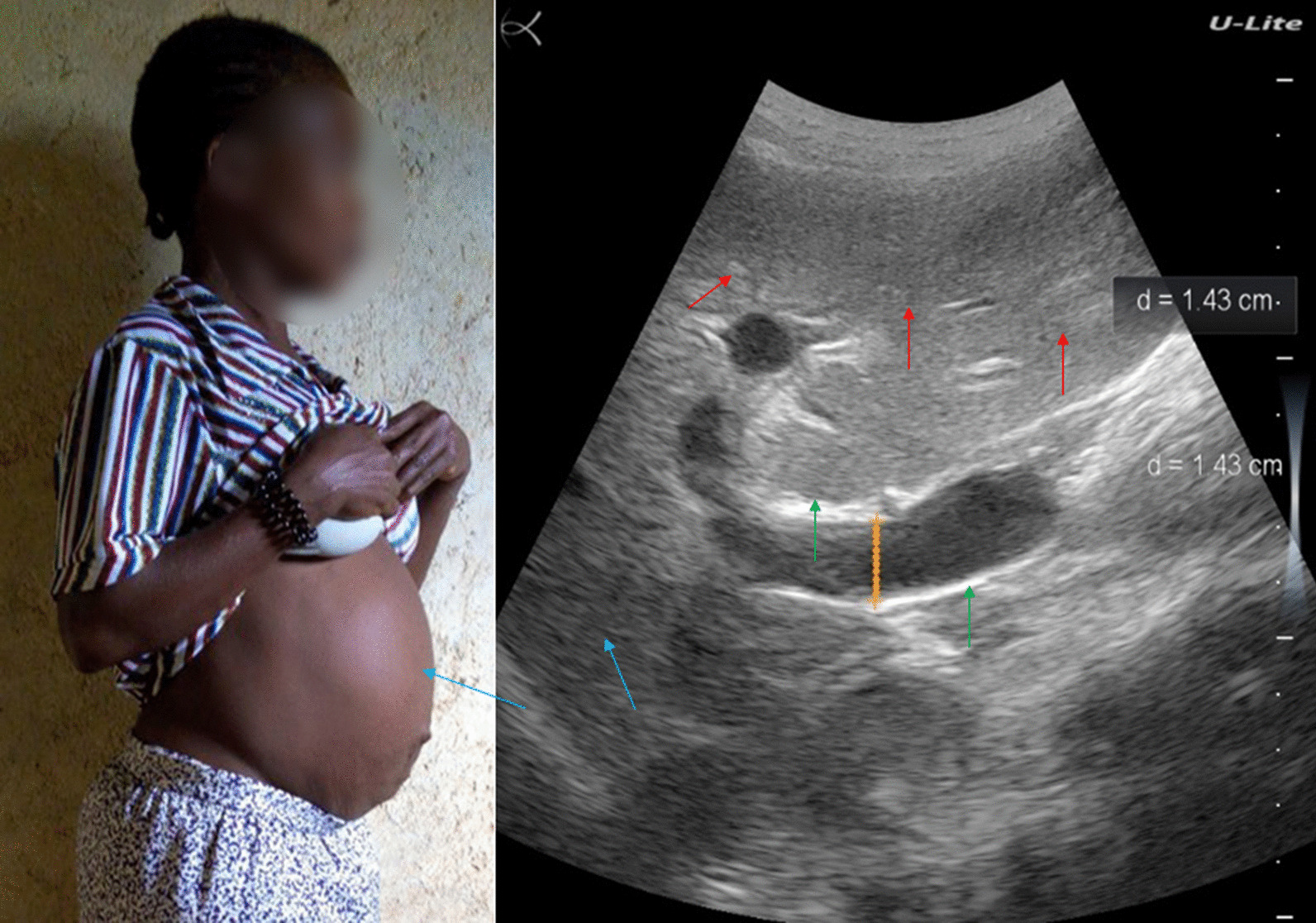
Patient #7, woman with a big belly (blue arrow, left). Ultrasound examination (right) showing liver with distorted architecture, nodular surface, heterogenous echotexture of the parenchyma (red arrows), decreased definition of the portal vein wall (green arrow), and ascites (blue arrow)

Her general state was poor. She had a highly distended abdomen, which showed epigastric tenderness and a detectable ascites during percussion (Fig. 7, left). Ultrasound examination showed a liver with distorted architecture, nodular surface, heterogenous echotexture of the parenchyma, decreased definition of the portal vein wall, ascites, and splenomegaly (Fig. 7, right).

A stool examination revealed a moderate-intensity *S. mansoni* infection (156 EPG). The POC-CCA was also positive. She received praziquantel treatment and was subsequently lost to follow-up.

### Patient 8

**Fig. 8:**
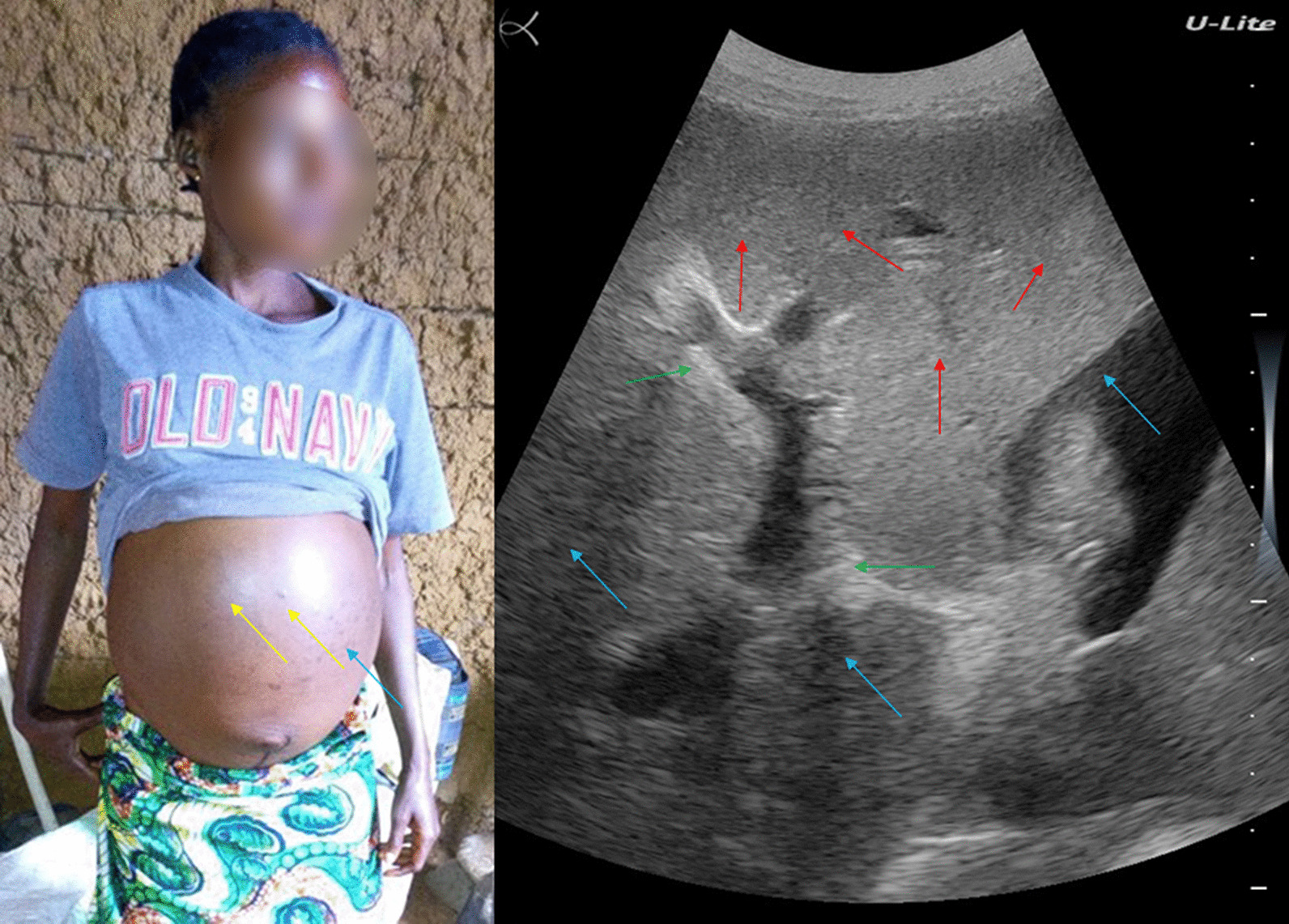
Patient #8, a young woman with a big belly, collaterals and abdominal rash spots (yellow arrows, left). Ultrasound examination (right) showing liver with distorted architecture, nodular surface, heterogeneous and starry night sky echotexture of the parenchyma (red arrows), decreased definition of the portal vein wall (green arrow), and ascites (blue arrow)

A 29-year-old farmer woman residing in the forest village of Pekele (Lolwa health district, southwest Ituri), a village opposite the Maholanzoka stream, presented with a highly distended belly, visible collaterals veins, and an abdominal rash (spots). Her general state was visibly poor (Fig. 8, left). She complained of weight loss, fatigue, abdominal pain, and diarrhea, without blood in the stool. She had not vomited blood before.

She had never heard of schistosomiasis and had not received treatment before.

Abdominal percussion showed dullness, revealing dramatic ascites. Ultrasound examination revealed a liver with distorted architecture, nodular surface, heterogeneous and starry night sky echotexture of the parenchyma, decreased definition of the portal vein wall, and splenomegaly (Fig. 8, right). All signs indicated a possible case of hepatosplenic schistosomiasis, even though both the Kato-Katz stool and POC-CCA urine examinations were negative for *Schistosoma* infection. She was later lost to follow-up.

## Discussion

Intestinal schistosomiasis is highly prevalent in sub-Saharan Africa. Most infections occur in poor, rural communities where daily intensive, sometimes professional, contact with natural water bodies is unavoidable. These communities are characterized by a general lack of access to safe water, inadequate sanitation facilities and poor-quality health services that are often unable to diagnose and treat prevailing health concerns. It is generally accepted that intestinal schistosomiasis is associated with a considerable degree of morbidity and mortality, and thereby poses a substantial individual and public health burden. Yet, the extent of severe disease burden among members of the most neglected communities is rarely explored and documented.

The detailed description of the health conditions of our eight patients with severe schistosomiasis document and visualize insights into the disease burden and the experiences the infection may pose. Our eight patients reported long-lasting intestinal symptoms consisting of recurring episodes of abdominal pain, diarrhea, and blood in the stool. Three patients reported severe weight loss and general fatigue. Hepatosplenomegaly indicated portal hypertension, which was confirmed by ultrasonography. Three patients had one or several episodes of hematemesis. Four patients were diagnosed with ascites, and an advanced-stage liver fibrosis was observed in five patients. One patient died shortly after having been seen.

The clinical observations of our patients are typical of those with chronic schistosomiasis mansoni. Six of the eight patients were diagnosed with *S. mansoni*. We could not confirm an *S. mansoni* infection in two patients with ascites. However, for several reasons, including multiple treatment with praziquantel, some patients suffering with intestinal schistosomiasis may be stool egg-negative [18]. One of the two egg-negative and CCA negative patients (Patient # 2) was treated with praziquantel several times.

Severe complications associated with *S. mansoni* may include upper digestive hemorrhaging with rapid blood loss, which can result in sudden death. A case series of 124 patients from Tanzania showed that during the chronic and terminal phase of intestinal schistosomiasis, patients with hepatosplenic involvement had a higher risk of dying from the disease [19]. The major cause of hematemesis is the rupture of esophageal varices associated with portal hypertension [20]. Whereas, ascites, an accumulation of circulatory fluids in the peritoneal cavity, is the result of anatomic and pathophysiologic abnormalities [21].

While our patients’ clinical picture is consistent with schistosomiasis mansoni disease development, we cannot exclude other etiologies that might have contributed to the symptoms and complications observed. For example, intestinal protozoan infections are frequent in tropical Africa and are a major contributor to poor gastrointestinal health. Viral hepatitis infections, which are of considerable prevalence in the DRC [22], may have also contributed to the morbidity observed. Likewise, *Plasmodium falciparum* is highly endemic to Ituri Province [23] and could have contributed to the development of splenomegaly observed among our patient group. As we could not assess all possible etiologies, there is a risk of overestimating the extent to which *S. mansoni* accounts for our patients’ disease patterns.

Three patients reported episodes of hematemesis. For patient #1, vomiting blood was the reason for his admission to hospital. While advanced schistosomiasis is one etiological factor of hematemesis, there are many others, such as gastric or duodenal ulcers, gastritis and esophagitis [19]. The use of non-steroidal, anti-inflammatory drugs was also identified as a contributing causal factor in a recent outbreak of hematemesis in Uganda [24]. However, among our patients, we diagnosed portal hypertension (enlarged portal vein diameter larger than 1.3 cm) and splenomegaly, both of which were found to be good predictors for active schistosomiasis as the etiological factor behind hematemesis [19].

Our patients resided in four villages that are highly endemic for *S. mansoni.* In a random sample (*n* = 164) of individuals six-years and older in these villages, almost 90% (87.1%) tested positive for an *S. mansoni* infection, either by Kato-Katz technique and/or the rapid POC-CCA urine test (Table 1). One third of the villagers examined reported diarrhea and/or blood in the stool. Upon abdominal ultrasound, more than one quarter were diagnosed with an enlarged liver, three quarters were diagnosed with splenomegaly and more than 70% had abnormal (fibrotic) liver parenchyma. The data demonstrate intensive transmission of *S. mansoni* and, hence, our eight patients are those with most severe outcomes—they represent the “tip of the iceberg” of the high degree of *S. mansoni* induced morbidity present in the community, and probably of numerous other patients with severe morbidity. Therefore, an intensification of public health interventions, i.e., preventive chemotherapy through mass-drug administration (MDA), is warranted.

Six of our patients resided in one of two villages (Gupe and Ndaro) on the shore of Lake Albert, where the transmission of *S. mansoni* is well known and documented. The patients from these villages knew about schistosomiasis and had previously been diagnosed and treated. The other two villages, Mandima and Pekele, are in southern Ituri and situated in a forested area. There, much less is known about the transmission of schistosomiasis. Neither of the two patients (patients #7 and #8) residing in these villages knew about schistosomiasis nor had they been diagnosed or treated before. Hence, information, education and communication measures are a vital part of any MDA campaign.

For more than two decades, Ituri Province has been subject to war, turmoil, and social conflict. The socioeconomic situation in Ituri is challenging, with a high degree of poverty and precarity. The DRC ranked 176/188 in the 2017 Human Development Index [8]. In 2011, a Water and Sanitation Program (WSP) strategic overview estimated that 50 million Congolese (75.0%) did not have access to safe water, while approximately 80–90% did not have access to improved sanitation [9]. Likewise, the United Nations International Children’s Emergency Fund (UNICEF)/WHO [10] 2017 database showed that in 2015, 84% of the DRC’s rural population had no hygiene facility, 45.3% had unimproved sanitation, 10.2% resorted to open defecation, 53% used unimproved water sources, and 16.0% used surface water. As for the country, Ituri Province is highly endemic for malaria and for viral, bacterial, protozoon, and helminth co-infections [4].

In DRC, there is a lack of information about schistosomiasis and other neglected tropical diseases [4]. However, in 2016 and 2017 we assessed *S. mansoni* infection and morbidity in 59 villages. We found high infection prevalence levels, particularly on the shores of Lake Albert and in the forest region [5]. In the latter area, schistosomiasis is largely unknown. Indeed, during colonial times, its prevalence was low (around 20%) [11]. In 2017, record rates of more than 90% were observed, along with hepatosplenomegaly rates of up to 25% [5].

In Ituri Province, ultrasound is a luxury health service. It is unavailable in most hospitals, including some general hospitals and clinics. Yet, ultrasound is an indispensable diagnostic tool that can assess hepatosplenic morbidity and guide the diagnosis and management of patients.

## Conclusions

Our clinical observations are limited to a few patients. However, they illustrate the severity of disease associated with *S. mansoni* infection in Ituri Province and provide further evidence of the severe neglect of the Ituri population in terms of access to adequate water, sanitation, and health services. The cases also present a strong argument for urgent and targeted interventions.

## Supplementary Information

**Additional file 1:** Individual questionnaire.

**Additional file 2:** Household questionnaire.

**Additional file 3: Figure S1.** Liver image patterns associated with schistosomiasis, by [12].

## Data Availability

Anonymized data can be obtained from the first authors upon reasonable request.

## Abbreviations

DRC: Democratic Republic of Congo
EPG: Eggs per gram
POC-CCA: Point-of-care circulating cathodic antigen
WHO: World Health Organization
