# Supplementary material for: Patients with severe schistosomiasis mansoni in Ituri Province, Democratic Republic of the Congo
Source: Infect Dis Poverty. 2021 Mar 25;10:39. doi: 10.1186/s40249-021-00815-6 (PMC7992822; doi:10.1186/s40249-021-00815-6)
Supplement: Supplementary file 1 — Additional file 1: Individual questionnaire. [file 40249_2021_815_MOESM1_ESM.docx]

**Individual Questionnaire**

| ENQUÊTE SCHISTOSOMIASE EN ITURI  QUESTIONNAIRE INDIVIDUEL | | | | | | | | | | | | |
| --- | --- | --- | --- | --- | --- | --- | --- | --- | --- | --- | --- | --- |
| Données générales | | | | | | | | | | | | |
| Date | _______/______/________ | | | | | | | | | | | |
| Numéro du ménage | \|____\| \|____\| \|____\| | | | | | | | | | | | |
| Numéro d’identification (ID) | \|____\| \|____\| \|____\| \|____\| \|____\| | | | | | | | | | | | |
| Village (nom) | ________________________________________ | | | | | | | | | | | |
| Données démographiques | | | | | | | | | | | | |
| Nom et postnom | ________________________________________ | | | | | | | | | | | |
| Date de naissance : | le ______/ _______/ ______________ | | | | | | | | | | | |
| Age | __________ | | | Sexe | | | _________ | | | | | |
| Statu matrimonial | Marié |  | Veuf | |  | | | Divorcé | |  | Polygame |  |
| Nationalité | **__________________** | | | | | Tribu | | | **_________________** | | | |
| Données anthropométriques | | | | | | | | | | | | |
| Poids | ______ kg (arrondi à 0,5 kg) | | | | | | | | | | | |
| Taille | ______ cm (arrondi à 0,5 cm) | | | | | | | | | | | |

| QUESTIONNAIRE INDIVIDUEL (suite) | | | | | |
| --- | --- | --- | --- | --- | --- |
| Données socio-économiques | | | | | |
| Occupation (cochez une ou plusieurs cases) | | | | | |
| Aucune |  |  | | | |
| Ménagère | Oui | |  | Non |  |
| Cultivateur | Oui | |  | Non |  |
| Pêcheur | Oui | |  | Non |  |
| Commercant | Oui | |  | Non |  |
| Enseignant(e) | Oui | |  | Non |  |
| Fonctionnaire | Oui | |  | Non |  |
| Infirmier(ère) | Oui | |  | Non |  |
| Médecin | Oui | |  | Non |  |
| Avocat | Oui | |  | Non |  |
| Pasteur | Oui | |  | Non |  |
| Autre (à préciser) | ___________________________________ | | | | |
| Education | | | | | |
| Primaire | Complète | |  | Incomplète |  |
| Secondaire | Complète | |  | Incomplète |  |
| Supérieur | Complète | |  | Incomplète |  |
| Universitaire | Complète | |  | Incomplète |  |
| Post-universitaire | Complète | |  | Incomplète |  |
| Religion | | | | | |
| Animiste | Oui | |  | Non |  |
| Catholique | Oui | |  | Non |  |
| Protestante | Oui | |  | Non |  |
| Reveil | Oui | |  | Non |  |
| Kimbanguiste | Oui | |  | Non |  |
| Musulman | Oui | |  | Non |  |
| Autre (à préciser) | ______________________________________________________ | | | | |

| QUESTIONNAIRE INDIVIDUEL (suite) | | | | | | |
| --- | --- | --- | --- | --- | --- | --- |
| Facteurs de risque : Pratiques (1) – Eau potable | | | | | | |
| Quelle eau buvez-vous ? (cochez une ou plusieurs cases) | | | | | | |
| L’eau du robinet de la maison | Oui |  | Non |  | De temps à temps |  |
| L’eau de la source aménagée du village | Oui |  | Non |  | De temps à temps |  |
| L’eau embouteillée | Oui |  | Non |  | De temps à temps |  |
| L’eau de notre puits privé | Oui |  | Non |  | De temps à temps |  |
| L’eau du puits de notre village | Oui |  | Non |  | De temps à temps |  |
| L’eau de la rivière | Oui |  | Non |  | De temps à temps |  |
| L’eau du lac | Oui |  | Non |  | De temps à temps |  |
| Autre (à préciser) | ___________________________________ | | | | | |
| Facteurs de risque : Pratiques (2) – Hygiène du corps | | | | | | |
| Vous baignez-vous le corps? | Oui |  | Non |  | De temps à temps |  |
| Si oui, où vous baignez-vous ? (cochez une ou plusieurs cases) | | | | | | |
| A la maison avec l’eau du robinet | Oui |  | Non |  | De temps à temps |  |
| A la maison avec l’eau de notre puits | Oui |  | Non |  | De temps à temps |  |
| Avec l’eau du puits du village | Oui |  | Non |  | De temps à temps |  |
| A la rivière | Oui |  | Non |  | De temps à temps |  |
| Au lac | Oui |  | Non |  | De temps à temps |  |
| A la source aménagée du village | Oui |  | Non |  | De temps à temps |  |
| Autre place (préciser) | ___________________________________ | | | | | |

| QUESTIONNAIRE INDIVIDUEL (suite) | | | | | | |
| --- | --- | --- | --- | --- | --- | --- |
| Hygiène des vêtements: Pratiques (2) – Eau de lessive, pêche, travaux de champs | | | | | | |
| Lavez-vous vos vêtements ? Oui Non De temps à temps (cochez une ou plusieurs cases) | | | | | | |
| Si oui, où les lavez-vous ? (cochez une ou plusieurs cases) | | | | | | |
| Avec l’eau du robinet de la maison | Oui |  | Non |  | De temps à temps |  |
| A la source aménagée du village | Oui |  | Non |  | De temps à temps |  |
| Avec l’eau de notre puits privé | Oui |  | Non |  | De temps à temps |  |
| Avec l’eau du puits de notre village | Oui |  | Non |  | De temps à temps |  |
| A la rivière | Oui |  | Non |  | De temps à temps |  |
| Au lac | Oui |  | Non |  | De temps à temps |  |
| Autre (à préciser) | ___________________________________ | | | | | |
| Facteurs de risque : Pratiques (3) – Autres contacts à risques | | | | | | |
| A part le bain et la lessive, quelle autre activité faites-vous dans l’eau de la rivière ou du lac ? | | | | | | |
| Faites-vous de la pêche ? | Oui |  | Non |  | De temps à temps |  |
| Si oui, à quelle fréquence ? Quotidienne Combien de fois par semaine ?______________ | | | | | | |
| Autre fréquence (préciser) | | | | | | |
| Faites-vous les travaux de champ ? | Oui |  | Non |  | De temps à temps |  |
| Si oui, avez-vous des jambières ? | Oui |  | Non |  |  |  |
| Portez-vous ces jambières au champ ? | Oui |  | Non |  | De temps à temps |  |

| QUESTIONNAIRE INDIVIDUEL (suite) | | | | | | |
| --- | --- | --- | --- | --- | --- | --- |
| Facteurs de risque : Pratiques (4) – Hygiène des mains | | | | | | |
| Lavage des mains (cochez une ou plusieurs cases) | | | | | | |
| Lavez-vous vos mains ? | Oui |  | Non |  | De temps à temps |  |
| Au savon ? | Oui |  | Non |  | De temps à temps |  |
| A la cendre ? | Oui |  | Non |  | De temps à temps |  |
| Si oui, quand vous lavez-vous les mains ? | | | | | | |
| Avant de manger | Oui |  | Non |  | De temps à temps |  |
| Après avoir été en toilette | Oui |  | Non |  | De temps à temps |  |
| Après avoir changé les couches de bébé | Oui |  | Non |  | De temps à temps |  |
| Autre (préciser) ___________ | Oui |  | Non |  | De temps à temps |  |
| Autre (à préciser) | ___________________________________ | | | | | |
| Facteurs de risque : Pratiques (5) – Port de chaussures | | | | | | |
| A -vous de chassures (fermées) ? | Oui |  | Non |  | Sandales |  |
| Si oui, les portez-vous ? (cochez une ou plusieurs cases) | | | | | | |
| Chaque fois que vous sortez ? | Oui |  | Non |  | De temps à temps |  |
| Si quelques fois, à quelles occasions les portez-vous ? (cochez une ou plusieurs cases) | | | | | | |
| Aller à l’église (mosquée) | Oui |  | Non |  | De temps à temps |  |
| Aller au marché | Oui |  | Non |  | De temps à temps |  |
| Aller à l’école | Oui |  | Non |  | De temps à temps |  |
| Autre occasion (préciser) | ___________________________________ | | | | | |

| QUESTIONNAIRE INDIVIDUEL (suite) | | | | | | |
| --- | --- | --- | --- | --- | --- | --- |
| Facteurs de risque : Pratiques (6) – Hygiène alimentaire | | | | | | |
| Comment avez-vous l’habitude de manger vos aliments ? (cochez une ou plusieurs cases) | | | | | | |
| Crus ? | Oui |  | Non |  | De temps à temps |  |
| Cuits ? | Oui |  | Non |  | De temps à temps |  |
| Autre (préciser) ______________________________________ | | | | | | |
| Si crus, quels sont les aliments que vous mangez crus ? Citez-les _________________________ | | | | | | |
| Les lavez-vous avant de manger ? | Oui |  | Non |  | De temps à temps |  |
| Après avoir été en toilette | Oui |  | Non |  | De temps à temps |  |
| Après avoir changé les couches de bébé | Oui |  | Non |  | De temps à temps |  |
| Autre (préciser) ___________ | Oui |  | Non |  | De temps à temps |  |
| Autre (à préciser) | ___________________________________ | | | | | |
| Facteurs de risque : Pratiques (7) – Autres habitudes dommageables pour la santé | | | | | | |
| Consommez-vous de l’alcool ou autres drogues ? (cochez une ou plusieurs cases) | | | | | | |
| Chaque fois que vous sortez ? | Oui |  | Non |  | De temps à temps |  |
| Si quelques fois, à quelles occasions les portez-vous ? (cochez une ou plusieurs cases) | | | | | | |
| Alcool ? | Oui |  | Non |  | De temps à temps |  |
| Si oui, depuis combien de temps ? ____________________________________ | | | | | | |
| Tabac ? | Oui |  | Non |  | De temps à temps |  |
| Si oui, depuis combien de temps ? ____________________________________ | | | | | | |
| Chanvre ? | Oui |  | Non |  | De temps à temps |  |
| Si oui, depuis combien de temps ? ____________________________________ | | | | | | |
| Essence ? | Oui |  | Non |  | De temps à temps |  |
| Si oui, depuis combien de temps ? ____________________________________ | | | | | | |
| Autre drogue ? (préciser) | Oui |  | Non |  | De temps à temps |  |
| Si oui, depuis combien de temps ? ____________________________________ | | | | | | |
| Facteurs de risque : Pratiques (8) – Durée de séjour au village | | | | | | |
| Depuis combien de temps résidez-vous ici ? ____________________________ (années) | | | | | | |

| QUESTIONNAIRE INDIVIDUEL (suite) | | | | | | |
| --- | --- | --- | --- | --- | --- | --- |
| Connaissances (1) Laisser la personne répondre librement et cocher ses réponses : | | | | | | |
| Connaissance sur la bilharziose comme maladie (cochez une ou plusieurs cases) | | | | | | |
| Avez-vous entendu parler de la bilharziose ? | Oui |  | Non |  |  | |
| Si oui, par quel canal de communication ? | | | | | | |
| Par la radio ? | Oui |  | Non |  |  | |
| En lisant un journal (livre) | Oui |  | Non |  |  | |
| Par les parents | Oui |  | Non |  |  | |
| A l’école | Oui |  | Non |  |  | |
| A l’église | Oui |  | Non |  |  | |
| A la mosquée | Oui |  | Non |  |  | |
| Par un ami ___________ | Oui |  | Non |  |  | |
| Par un autre moyen (à préciser) | ___________________________________ | | | | | |
| Si oui, comment ses manifeste-t-elle ? (signes et symptômes) | | | | | | |
| Par la diarrhée | Oui |  | Non |  | Je ne sais pas |  |
| Selles avec du sang | Oui |  | Non |  | Je ne sais pas |  |
| Mucus dans les selles | Oui |  | Non |  | Je ne sais pas |  |
| Gros foie | Oui |  | Non |  | Je ne sais pas |  |
| Grosse rate | Oui |  | Non |  | Je ne sais pas |  |
| Ventre rempli de liquide | Oui |  | Non |  | Je ne sais pas |  |
| Vomissement de sang | Oui |  | Non |  | Je ne sais pas |  |
| Autre signe ou symptôme (préciser) | ___________________________________________ | | | | | |
| Quelle en est la cause ? (Cause) | | | | | | |
| Je ne sais pas | | | | | | |
| Sorcellerie | Oui |  | Non |  | Je ne sais pas |  |
| Microbes | Oui |  | Non |  | Je ne sais pas |  |
| Vers intestinaux | Oui |  | Non |  | Je ne sais pas |  |
| Autre vers (préciser) | ___________________________________________ | | | | | |
| Autre cause (préciser) | ___________________________________________ | | | | | |

| Comment contracte-t-on la bilharziose ? (Transmission) | | | | | | |
| --- | --- | --- | --- | --- | --- | --- |
| Je ne sais pas | | | | | | |
| En buvant de l’eau contaminée | Oui |  | Non |  | Je ne sais pas |  |
| En jouant ou en travaillant dans l’eau | Oui |  | Non |  | Je ne sais pas |  |
| En marchant pieds nus | Oui |  | Non |  | Je ne sais pas |  |
| Par le tatouage | Oui |  | Non |  | Je ne sais pas |  |
| En saluant les personnes atteintes | Oui |  | Non |  | Je ne sais pas |  |
| Par les relations sexuelles | Oui |  | Non |  | Je ne sais pas |  |
| Autre mode de transmission (préciser) | ___________________________________________ | | | | | |
| Comment peut-on éviter la bilharziose ? (Prévention) | | | | | | |
| Je ne sais pas | | | | | | |
| En buvant de l’eau potable | Oui |  | Non |  | Je ne sais pas |  |
| En ne jouant ni travaillant dans l’eau | Oui |  | Non |  | Je ne sais pas |  |
| En portant des jambières en cultivant | Oui |  | Non |  | Je ne sais pas |  |
| En portant des chaussures fermées | Oui |  | Non |  | Je ne sais pas |  |
| En lavant les vêtements dans une eau saine | Oui |  | Non |  | Je ne sais pas |  |
| En utilisant les préservatifs | Oui |  | Non |  | Je ne sais pas |  |
| Autre mode de prévention (préciser) | ___________________________________________ | | | | | |
| Comment traite-t-on la bilharziose ? (Traitement) | | | | | | |
| Je ne sais pas | | | | | | |
| En prenant le Praziquantel (Biltricide) | Oui |  | Non |  | Je ne sais pas |  |
| En prenant le mebendazole ou l’albendazole | Oui |  | Non |  | Je ne sais pas |  |
| Autre mode de médicament (préciser) | | | | | | |
| Attitudes et histoire familiale – 1 (laisser la personne répondre librement et cocher ses réponses) | | | | | | |
| Y a-t-il quelqu’un de votre famille qui a été diagnostiqué de bilharziose | Oui |  | Non |  | Je ne sais pas |  |
| Si oui, qu’avez-vous fait pour lui ? (Prise en charge) | | | | | | |
| Rien | | | | | | |
| Médicaments traditionnels | Oui |  | Non |  | Je ne sais pas |  |
| Aller consulter les tradi-praticiens | Oui |  | Non |  | Je ne sais pas |  |
| Acheter les médicaments à la pharmacie | Oui |  | Non |  | Je ne sais pas |  |
| Aller au Centre de santé | Oui |  | Non |  | Je ne sais pas |  |

| Cas de décès dû à la bilharziose ? (Histoire familiale – 2) | | | | | | |
| --- | --- | --- | --- | --- | --- | --- |
| Y a-t-il eu quelqu’un de votre famille mort de bilharziose ? | Oui |  | Non |  | Je ne sais pas |  |
| Je ne sais pas | | | | | | |
| Pensez-vous que la bilharziose est un grand problème dans votre village ? | Oui |  | Non |  | Je ne sais pas |  |
| Si oui, que devriez-vous faire pour lutter contre la bilharziose ? (Réponse libre) | | | | | | |
| _____________________________________________________________ | | | | | | |
| MERCI D’AVOIR REPONDU A NOS QUESTIONS | | | | | | |
