# Supplementary material for: Patients with severe schistosomiasis mansoni in Ituri Province, Democratic Republic of the Congo
Source: Infect Dis Poverty. 2021 Mar 25;10:39. doi: 10.1186/s40249-021-00815-6 (PMC7992822; doi:10.1186/s40249-021-00815-6)
Supplement: Supplementary file 2 — Additional file 2: Household questionnaire. [file 40249_2021_815_MOESM2_ESM.docx]

**Household Questionnaire**

| ENQUÊTE SCHISTOSOMIASE EN ITURI  QUESTIONNAIRE MENAGE |
| --- |
| Date_______/______/________  Numéro d’identification (ID) (ménage) \|____\| \|____\| \|____\|  Village (nom)_______________________________________________________________  Chef du ménage  Nom et postnom ____________________________________________________________________  Homme Femme  Habitation :   - Mur –  :   En paille ou en feuilles  En terre battue Crépi Non crépi Avec peinture  En pierre Crépi Non crépi Avec peinture  En briques adobe non-cuites Crépi Non crépi Avec peinture  En briques adobe cuites Crépi Non crépi Avec peinture  En briques pressées cuites Crépi Non crépi Avec peinture  En monobloc en ciment Crépi Non crépi Avec peinture   - Plancher –  :   En terre battue Pavé en argile Non pavé  En briques adobe non-cuites  En briques adobe cuites  En briques pressées cuites  Pavé en ciment Avec peinture Sans peinture  En carreaux  En granitos coulés   - Toit – :   En chaume ou en feuilles  En bâche  En tôles ondulées BWG 30 – 36  En tuiles  En ardoise  En tôles ondulées BWG 20 – 28  Distance par rapport au plan d’eau le plus proche ____________________________________________  Revenu annuel moyen du ménage (estimation) : _____________ USD  Elevage au ménage :  Aucun  Poules ? Oui Non Combien ? ______________________________  Canard ? Oui Non Combien ? ______________________________  Chèvres ? Oui Non Combien ? ______________________________  Brebis ? Oui Non Combien ? ______________________________  Porcs ? Oui Non Combien ? ______________________________  Chiens ? Oui Non Combien ? ______________________________  Chats ? Oui Non Combien ? ______________________________  Vaches ? Oui Non Combien ? ______________________________  Autre animal (préciser) Oui Non Combien ? ______________________________  Objet possédé :  Aucun  Vélo Oui Non Combien ? ______________________________  Machine à coudre Oui Non Combien ? ______________________________  Frigo Oui Non Combien ? ______________________________  Congélateur Oui Non Combien ? ______________________________  Moto Oui Non Combien ? ______________________________  Motopompe Oui Non Combien ? ______________________________  Voiture Oui Non Combien ? ______________________________  Camion Oui Non Combien ? ______________________________  Autre (à préciser) _____________________ Oui Non Combien ? ____________________________    Facteurs de risque :   - Accès à l’eau potable –   Où vous approvisionnez-vous en eau potable ?  L’eau du robinet de la maison  L’eau embouteillée  L’eau de notre puits privé  L’eau de la source aménagée du village Distance ______________________________ km  L’eau du puits du village Distance ______________________________ km  L’eau de la rivière Distance ______________________________ km  L’eau du lac Distance ______________________________ km  Autre eau (préciser) _____________________ Distance ______________________________ km   - Assainissement –   Latrine dans votre parcelle ? Présence (vérifier) Oui Non  Si latrine présente, vérifier qualité de la latrine :  Plancher Mauvaise Bonne  Porte Mauvaise Bonne  Profondeur Mauvaise Bonne  Propreté orifice Mauvaise Bonne  Mouches Mauvaise Bonne  Distance < 10 m = Mauvaise > 10 m = Bonne  Utilisation – L’utilisez-vous ? Oui Non Quelques fois  Quand la dernière fois avez-vous utilisé la latrine ? ______________________________________  Si vous n’utilisez pas de latrine, Où allez-vous faire vos besoins ?  Derrière la maison Oui Non Quelques fois  Dans la brousse proche Oui Non Quelques fois  Dans la rivière Oui Non Quelques fois  Dans le lac Oui Non Quelques fois  Dans le seau Oui Non Quelques fois  Dans les sachets Oui Non Quelques fois  Autre place (préciser) ____________________________________  Trou à ordures :  Présence : Absent Présent  Qualité : Mauvaise Bonne |
| Quel est, selon vous, le plus grand problème de santé de votre village ?  _____________________________________________________________________________________  Selon vous, qu’est-ce qui doit être fait pour résoudre ce problème dans votre village ? ________________________________________________________________________________  Par qui ? _________________________________________________________________________  Comment ? ________________________________________________________________________  A quel moment (Quand) ? _____________________________________________________________  Par quel moyen ? ____________________________________________________________________ |
