# Supplementary material for: Patients with severe schistosomiasis mansoni in Ituri Province, Democratic Republic of the Congo
Source: Infect Dis Poverty. 2021 Mar 25;10:39. doi: 10.1186/s40249-021-00815-6 (PMC7992822; doi:10.1186/s40249-021-00815-6)
Supplement: Supplementary file 3 — Additional file 3: Figure S1. Liver image patterns associated with schistosomiasis, by [12]. [file 40249_2021_815_MOESM3_ESM.docx]

**Additional information: Figure**


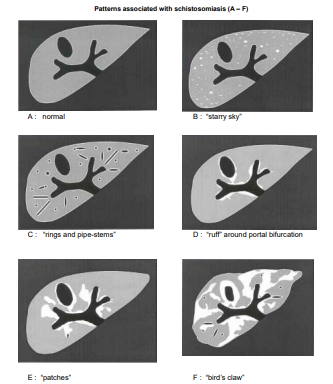


**Figure S1:** Liver image patterns associated with schistosomiasis, by [15].
